# Resistance to Arsenic- and Antimony-Based Drugs

**DOI:** 10.1155/S1565363303000153

**Published:** 2003

**Authors:** Milena Salerno, Arlette Garnier-Suillerot

**Affiliations:** Laboratoire de Physicochimie Biomoléculaire et Cellulaire (LPBC/CSSB UMR 7033) Université Paris Nord 74 rue Marcel Cachin Bobigny 93017 France

## Abstract

Organic arsenicals were the first antimicrobial agents specifically synthesized for the treatment of infectious diseases such as syphilis and sleeping sickness. For the treatment of diseases caused by trypanosomatid parasites, organic derivatives of arsenic and the related metalloid antimony are still the drugs of choice. Arsenic trioxide, As203, has been used for a long time in traditional Chinese medicines for treatment of various diseases, and it has recently been shown to be clinically active in acute promyelocytic leukemias. Resistance to metalloid salts is found in bacteria, fungi, parasites and animals. In some organisms, resistance involves overproduction of intracellular thiols. In many cases, resistance to arsenic salts is the result of removal of the metalloid from the cytosol usually by extrusion from the cell. In eukaryotes resistance to arsenic and antimony is conferred by membrane transport proteins of the MRP family. The human MRP1, a member of this family, is frequently amplified in cancer cells and it is well-documented that MRPl-overexpressing cells poorly accumulate arsenic and antimony because of enhanced cellular effiux which depends on the presence of GSH.


### Resistance to Arsenic- and Antimony-Based Drugs

Milena Salerno and Arlette Garnier-Suillerot*

*Laboratoire de Physicochimie Biomoléculaire et Cellulaire (LPBC/CSSB UMR 7033)*
*Université Paris Nord, 74 rue Marcel Cachin, 93017 Bobigny, France*

(Received January 13, 2003; Accepted January 22, 2003)

### ABSTRACT

Organic arsenicals were the first antimicrobial agents specifically synthesized for the treatment of infectious diseases such as syphilis and sleeping sickness. For the treatment of diseases caused by trypanosomatid parasites, organic derivatives of arsenic and the related metalloid antimony are still the drugs of choice. Arsenic trioxide, $As_2O_3$, has been used for a long time in traditional Chinese medicines for treatment of various diseases, and it has recently been shown to be clinically active in acute promyelocytic leukemias. Resistance to metalloid salts is found in bacteria, fungi, parasites and animals. In some organisms, resistance involves overproduction of intracellular thiols. In many cases, resistance to arsenic salts is the result of removal of the metalloid from the cytosol usually by extrusion from the cell. In eukaryotes resistance to arsenic and antimony is conferred by membrane transport proteins of the MRP family. The human MRP1, a member of this family, is frequently amplified in cancer cells and it is well-documented that MRP1-overexpressing cells poorly accumulate arsenic and antimony because of enhanced cellular efflux which depends on the presence of GSH.

### INTRODUCTION

The introduction of antibiotics in the 1940s opened up a completely new prospect to medical science and the treatment of infectious diseases. A broad variety of drugs active against several infectious organisms were discovered or developed. However, the widespread, and sometimes uncontrolled, use of these drugs has led to the emergence of defense mechanisms that, at present, are the major drawback to the drug-based treatment of infectious diseases and cancers. Most strikingly, such resistance is not restricted to the drugs (or analogs) used in the treatment but also involves several structurally and functionally unrelated compounds. This

* Corresponding author

Telephone: 33 1 48 38 77 48.

Fax: 33 1 48 38 77 77.

E-mail: garnier@lpbc.jussieu.fr.

phenomenon, which has been termed multidrug resistance (MDR) can be caused by various mechanisms and is known to play an important role in the drug resistance of a broad range of pathogenic bacteria, parasitic protozoa, and tumor cells. Altogether, these diseases are responsible for millions of deaths yearly /1-4/.

Multidrug resistance (MDR) in tumour cells is often caused by the overexpression of two transporters the P-glycoprotein (P-gp) and the multidrug resistance associated protein (MRP1). Both proteins belong to the superfamily of the so-called ATP binding cassette transport proteins (ABC transporters), which are known to be dependent on ATP hydrolysis for the translocation of substrate across membranes /5, 6/.

P-gp is an unusual ABC protein in that it appears to be highly promiscuous: hundreds of compounds have been identified as substrates. The spectrum of MDR compounds includes a large number of anticancer drugs (e.g., anthracyclines, vinca alkaloids, taxanes) as well as steroids, fluorescent dyes, rhodamine, and the γ-emitting radiopharmaceutical $99m_{Tc\text{-}MIBI}$ /1,2/. P-gp can transport neutral and positively charged molecules but not negatively charged ones.

MRP1 belongs to a second class of transporters whose mechanism of action differs from that of P-gp-class transporters. MRP1 can transport a large range of drugs conjugated to negatively charged hydrophilic ligands such as glutathione, glucuronic acid, and sulfate /7-9/. It can also transport arsenite and antimonite when GSH is present. MRP1 is also able to confer resistance to drugs that are not known to be conjugated to any acidic ligand, such as doxorubicin or the vinca alkaloids. Such resistance seems to require the presence of glutathione, and available evidence indicates that MRP1 can co-transport drug with glutathione and even glutathione alone can be transported, though at a lower rate /7, 10-12/. MRP1 can also transport neutral, as well as positively negatively charged, molecules.

In summary, a clear distinction in the mechanism of translocation of substrates by MRP1 or P-gp is indicated by the finding that, in most cases, the MRP1-mediated transport.of substrates is inhibited by depletion of intracellular glutathione (GSH), which has no effect on their P-gp-mediated transport. In this small review we will focus on the resistance to arsenic- and antimony-based drugs.

### ARSENIC: TOXICITY AND METABOLISATION

Arsenic is synonymous with poison and geological sources of inorganic arsenic are environmental problems worldwide. This metalloid is ubiquitously present in the environment, where it occurs mainly as compounds of As(III) and As(V) ions. Its toxicity makes it a useful ingredient in herbicides and pesticides Epidemiological studies have shown that chronic exposure to inorganic arsenic : arsenite, $As(III)O_3^{3-}$, and arsenate, $As(V)O_4^{3-}$, result in liver injury, peripheral neuropathy, and an increased incidence of cancer of the lung, skin, bladder, and liver /13-16/. In addition, occupational exposure to arsenic increases the lung cancer incidence among smokers and underground miners in a manner consistent with a synergistic interaction of arsenic with tobacco smoke or radon found in mines.

Arsenite and arsenate are extensively metabolized by humans and many other species to yield four major methylated metabolites, $CH_3As(V)O_3^{2-}$, $CH_3As(III)O_2^{2-}$, $(CH_3)_2As(V)O_2^-$ and $(CH_3)_2As(III)O^-$. Hence, humans exposed to inorganic arsenic by ingestion or inhalation excrete inorganic arsenic, methyl As and dimethyl As in urine /17-19/. Cullen *et al.* /20/ have provided a comprehensive scheme for the metabolism of arsenic that can be summarized as follows:

$As(V)O_4^{3-} + 2e + 2H^+ \rightarrow As(III)O_3^{3-} + H_2O$

$As(III)O_3^{3-} + CH_3^+ \rightarrow CH_3As(V)\ O_3^{2-}$

$CH_3As(V)\ O_3^{2-} + 2e + 2H^+ \rightarrow CH_3As(III)\ O_2^{2-} + H_2O$

$CH_3As(III)\ O_2^{2-} + CH_3^+ \rightarrow (CH_3)_2\ As(V)\ O_2^-$

$(CH_3)_2As(V)\ O_2^- + 2e + 2H^+ \rightarrow (CH_3)_2\ As(III)\ O^- + H_2O$

Here, the reduction of arsenic from pentavalency to trivalency is a prerequisite for its oxidative methylation, suggesting both trivalent and pentavalent metabolites to be intermediates or final products in this metabolic pathway. Methylated arsenicals have unique biological effects. At high dose levels they are tumor promoters and carcinogen. Most data suggest that the pathway for the methylation of arsenic, which produces methylated arsenicals containing As(V), should be regarded as a mechanism for the activation, not for the detoxification, of arsenic. Therefore, methyl As and dimethyl As are likely to be the metabolites of arsenic that account for at least some of the adverse effects associated with chronic exposure to arsenic /21-23/.

A central issue in arsenic biochemistry is the role of sulfhydryl-containing molecules and especially of gluthathione (GSH) because the reduction of As(V) may use glutathione as a source of reducing equivalents /24, 25/; it can also be catalyzed by As(V) reductases /26, 27/. GSH is a tripeptide, (γ-L-Glutamyl-L-Cysteinylglycine) and a potentially polydentate ligand present in many cells at millimolar concentration. Generally it is the most abundant nonprotein thiol. GSH is known to bind a variety of essential and non-essential metals, playing critical roles in the cellular and systemic metabolism of these metals /28/. The formation of the As(III)-GSH complexes have been thoroughly studied /24, 25/. It has been shown that $As(GS)_3$ is formed and that the binding site is the cysteinyl sulfydryl. However, it must be pointed out that these experiments have been performed at acidic pH value (~3) and that about six hours are required for the complete formation of the complex /25, 29/. The kinetics of formation of $As(GS)_3$ is therefore very slow at pH 3 and even slower at pH 7.3. According to the data of Delnomdedieu *et al.* /25/, the $As(GS)_3$ complex, which has been prepared at acidic pH value, is stable over the pH range from 1.5 to 7.0 - 7.5 and rather unstable above pH 7.5. Thus, there is little chance to observe its major formation when As(III) and GSH are mixed at pH higher than 7.

Actually, with one exception /30/, the formation of arsenic-glutathione complex has not been demonstrated *in vivo.* Thus only Kala *et al.* /30/ have demonstrated the presence of As(III)-glutathione complex in the bile of rats injected with arsenic. They have observed that when rats are intravenously treated with sodium arsenite at high doses (5mg/kg i.e. ~300µM arsenic), arsenic is recovered in the bile complexed to GSH. However, when lower doses, that are relevant to human exposure (0.1 - 0.5 mg/kg i.e. ~ 5 - 30 µM) are used, arsenic complexed to GSH was almost undetectetable. According to these authors, a likely explanation for this finding is that $As(GS)_3$ is found only at high doses (5mg/kg) because the ability of cells to methylate arsenic is saturated, whereas at lower doses all arsenic can be methylated. Under experimental conditions similar to those used by Kala *et al.* /30/, Gregus *et al.* /31/ have not detected As-GSH species and have found that monomethylarsenous acid is the major biliary.metabolite in rats. Therefore, it must be kept in mind that the major human metabolic pathway for arsenic is methylation /30, 32/.

### MECHANISM OF DETOXIFICATION IN DIFFERENT ORGANISMS.

It is likely that life evolved in waters rich in dissolved metals and metalloids, such as arsenic. Therefore all organisms, prokaryotic, eukaryotics or archea have evolved resistance to drugs and toxic metal. Not surprisingly, resistance to metalloid salts is found in bacteria, fungi, parasites and animals. In some organisms, resistance involves overproduction of intracellular thiols /33/ but in many cases, resistance to arsenic salts is the result of removal of the metalloid from the cytosol usually by extrusion from the cell /34, 35/. In *Escherchia coli*, high level resistance to salts of the trivalent metalloids arsenite and antimonite is conferred by the presence of plasma membrane protein ArsAB which pumps out the metalloid oxyanions. This pump does not belong to the ABC transporter family /35/. The ArsAB pump is composed of ArsA and ArsB. ArsB is the 45-kDa membrane sector of the pump, and has 12 membrane spanning segments. ArsA, is a 63-kDa protein with two homologous halves and is the catalytic subunit, exhibiting As(III)/Sb(III)-stimulated ATPase activity. Thus, the complex of ArsA and ArsB forms an anion-translocating ATPase that calalyzes extrusion of arsenite or antimonite.

Up to now no eukariotic ArsAB orthologs have been identified. However, another two families of membrane proteins (see below) have eukaryotic representatives that have been shown to produce arsenite and antimonite resistance: the ACR family and the MRP family.

### ORGANIC DERIVATIVES OF ARSENIC AND ANTIMONY FOR THE TREATMENT OF DISEASES CAUSED BY PARASITES.

Organic arsenicals were the first antimicrobial agents specifically synthesized for the treatment of infectious diseases. Paul Ehrlich won the Nobel Prize in 1908 for synthesis of the arsenical salvarsan, his "silver bullet" developed for treatment of syphilis and sleeping sickness. For treatment of diseases caused by trypanosomatid parasites, organic derivatives of arsenic and the related metalloid antimony are still the drugs of choice /3/.

Parasites of the genus *Leishmania* are distributed worldwide, and 10-15 million people are estimated to be infected with 400,000 new cases each year /36/. *Leishmania* are endemic from the USA-Mexico border through all of Central America to several of the South American countries, around the Mediterranean sea, in the North and East Africa, in Southern Russia, the Middle East, India and China. Cases of *Leishmania* infections are rising even in developed countries. Increases in traveling have raised the number of *Leishmania* in non-endemic areas. Incidence of leishmaniasis is rising also because of the lack of vaccines, difficult vector control and increase in resistance to chemotherapy. Leishmania has also emerged as a serious opportunistic pathogen in HIV-infected humans. The life cycle of the parasite is relatively simple. Flagellated promastigotes are transmitted to humans by the phlebotomine female sandfly. Parasites are engulfed by macrophages, where the promastigotes differentiate into aflagellated amastigotes within the phagolysosomes of the host macrophages. Upon *Leishmania* infection, several macrophage functions are altered, allowing parasites to replicated and escape the host defense mechanisms /37/.

The only effective way to control *Leishmania* infections currently is chemotherapy. The first agents with a favorable therapeutic index, the pentavalent antimonials, were introduced in the 1940s and are still the mainstay of therapy to all forms of leishmaniasis. Thus, the treatment of choice of human visceral leishmaniasis is the administration of pentavalent antimony (Sb (V)) containing drugs such as sodium stibogluconate (Pentostam; Wellcome, UK), in which antimony is complexed with gluconic acid or meglumine antimonate (glucantime; Rhone-Poulenc France) in which antimony is complexed with the sugar meglumine. The treatment of leishmaniasis with antimonial regimens is complicated by drug toxicity (cardiac and renal) and by drug resistance. Despite the fact that antimony-containing drugs have been used in antiprotozoan therapy for over half a century, little is known about its biological properties and mechanisms of toxicity. Pentavalent antimony (Sb(V)) inhibits glucose catabolism and ATP formation via the glycolytic pathway and fatty acid Β-oxidation in *Leishmania mexicana* /38/. Furthermore, Sb(V) can form complexes with various carbohydrates. Trivalent antimony /Sb(III)/ appears to interact with key sulfhydryl groups of leishmanial proteins, thereby probably causing enzyme inhibition /39/.

As seen earlier, bacteria and yeast that live in environments contaminated with arsenate reduce the element intracellularly to arsenite as part of the mechanism that was evolved to evade the toxic effects of this heavy metal /35/. Given that *Leishmania* spp. are not necessarily exposed to heavy metals in their natural habitats (sandflies and vertebrates), they may not have developed this type of protective mechanism. In fact, by reducing nontoxic Sb(V), *Leishmania* actually expose themselves to the lethal effects of Sb (III).

Actually, many patients fail to respond to the treatment of drugs containing antimony, because parasites resistant to these drugs are now becoming widespread /40, 41/. *Leishmania* strains resistant not only to pentavalent antimony but also to trivalent antimony and arsenite have been described /42/.

One mechanism of resistance recently described is the inability of the parasite to reduce Sb(V) to Sb(III) /43/. Stage-specific intracellular Sb(V) reducing activity was apparent in amastigotes which reduced the negligibly toxic Sb(V) to highly toxic Sb(III). This amastigote-specific reducing activity was deficient in the pentostam-resistant mutant *L. Donovani* Ld1S.20. These data indicate that Sb(V) anti-leishmanial activity is dependent on its reduction to Sb(III). The mechanism of this novel intracellular Sb(V) reduction has yet to be identified, and it may or may not be enzymatic.

Amplification of the P-gp A and gsh1 genes is frequently observed in arsenite- and antimonite-tolerant *Leishmania* cells. gsh1 encodes γ-glutamulysteine synthetase, an enzyme involved in the rate-limiting step in glutathione biosynthesis. Pgp A is homologous to human MRP1 /42/ and most likely confers resistance by sequestering metal-thiol conjugates into an intracellular vesicle /44/. These studies point to a key role of transporters and thiol levels in metal resistance in *Leishmania*.

### ARSENIC TRIOXIDE AND THE TREATMENT OF ACUTE PROMYELOCYTIC LEUKEMIA.

Cancer itself is a major worldwide health concern, with an estimated 6 millions new cases per year. Until now, the only possible methods of curing systemic cancers, such as leukemia, lymphoma, and unifocal tumors that have spread by metastasis, have involved systemic treatments such as chemotherapy and immunotherapy.

Arsenic compounds have been used in the treatment of leukemia, particularly in chronic myeloid leukemia (CML), erythremia, and Hodgkin's lymphoma since 1865 in the form of Fowler solution (potassium arsenite, $KAsO_2$). An arsenic compound in the Ayurvedic pharmacopeia of India /45/ was recognized as useful to control blood cell counts in patients with CML over 100 years ago. Two arsenic compounds have been utilized in Chinese traditional medicine for more than 500 years. One is *Pishang*, or white arsenic, essentially containing arsenic trioxide ($As_2O_3$), which is still used clinically in the treatment of certain skin diseases and asthma and to promote the healing of surgical wounds. The other compound is *Xiong-huang* (or realgar compound), which contains arsenic disulfide and was administrated in the treatment of CML for more than 40 years. However, Fowler solution was abandoned in the treatment of CML due to its chronic toxicity with long-term use and the discovery of more effective chemotherapeutic agents. Arsenic trioxide, $As_2O_3$, has been used for a long time in traditional Chinese medicines for treatment of various diseases, and it has.recently been shown to be clinically active in acute promyelocytic leukemias /46/.

Now the arsenic trioxide $As_2O_3$ is used for the treatment of acute promyelocytic leukemia (APL). This disease is one of many subtypes of acute myelogenous leukemia, so named because the leukemic cells are "frozen" at a stage that mimics the appearance of normal promyelocytes in the marrow. Specifically, the $As_2O_3$ is used for the treatment of patients with acute APL who have not responded to, or have relapsed following the use of all trans-retinoic acid and anthracycline-based chemotherapy, which is considered first line therapy. Exactly how $As_2O_3$ mediates its clinical efficacy is not fully understood. Two main mechanisms of action of $As_2O_3$ have been identified: promotion of APL cell differentiation and induction of apoptosis /47/. At concentrations in the range 1-2 μM, $As_2O_3$ induces apoptosis and, clinically, during $As_2O_3$ treatment, chromatin of leukemic cells becomes dense and coarse, and apoptotic bodies are seen. The mechanism of apoptosis induction by $As_2O_3$ at high concentrations is primarily through disruption of the mitochondrial transmembrane potential

Although $As_2O_3$ exhibits an anticancer effect against a broad spectrum of cancer cell lines, there have been few reports of its efficacy in clinical trials, apart from the high remission rate achieved in APL.

Transfection of human tumor cells with a multidrug resistant-associated protein (MRP1) gene has shown a cross-resistance to arsenite /48, 49/, which may limit the clinical use of this anticancer drug for treatment of MRP1-positive tumors /50, 51/ and it is now well-documented that MRP1-overexpressing cells poorly accumulate arsenic and antimony because of enhanced cellular efflux which depends on the presence of GSH /50-52/.

The presence of glutathione is required for the MRP1-mediated efflux of arsenic and antimony, however, different hypothesis can be made: i) the metalloid forms a complex with GSH and this complex is pumped out by MRP1, ii) the metalloids are co-transported with GSH, as this is the case for daunorubicin /10, 12/ and vincristine /53/. It follows that one of the questions that have to be solved here is: what is the chemical nature of the arsenic and antimony species which are pumped out via MRP1 protein.

Based on the following observations, the formation and the transporter-mediated efflux of As-GSH complex has often been postulated. It has been shown that either As(V) or As(III) treatment induced increased biliary excretion of non-protein thiols in the rat /54/. An increased biliary excretion of glutathione is also generated by the glutathione-dependent hepatobiliary transport of antimony and bismuth /55/. Because this enhancement was not observed when the thiol groups were blocked by a firmly bound metal ion (e.g. Hg(II)), and based on the close chemical similarity of the trivalent antimony, bismuth and arsenic, the authors hypothesized that generation of biliary GSH by arsenic, antimony and bismuth is due to hepatic formation, biliary excretion and subsequent decomposition of unstable arsenic-GSH, antimony-GSH and bismuth-GSH complexes. Because the pH of bile is neutral or slightly alkaline, this proposition is in agreement with the possible dissociation of the GSH-As complexes observed at pH>7.5 /25/.

In MRP1-overexpressing cells, Zaman *et al.* /56/ have observed that MRP1 increases the export of glutathione from the cell and this increase is further elevated in the presence of arsenite. They subsequently proposed that $As(GS)_3$ complex may be the form in which arsenite is excreted. Thus, the significance of the As(III)-glutathione complexes in transport and metabolism *in vivo* is still unknown and requires further studies /24/.

To develop a better understanding of arsenic and antimony excretion by MRP1-overpressing cells, we have recently measured the kinetic parameters for the uptake and release of these metalloids in MDR and parental cancer cells /29/ One question is: what are the arsenic and antimony species which diffuse passively through the plasma membrane? As(III) and Sb(III) are semi metals that can form either oxyanions or soft metal covalent bonds with the thiolate groups. The chemistry of arsenic in water is not simple but there is little doubt that at pH 7, aqueous solutions of inorganic arsenites contains $As(OH)_3$ pK1 = 9.2 /20/. It follows that the uptake of arsenic in the cells occurs by passive diffusion of the neutral trihydroxide. Now, once inside the cells, what is the evolution of $As(OH)_3$. The data obtained with sensitive cells are very important in answering this question. Actually, the observation that, when cells are incubated with $As_2O_3$, at the pseudo steady state obtained after 1h, the extra-and intracellular concentrations of arsenic are the same, indicates that there is a transmembrane equilibrium of the species that diffuses passively through the membrane; this means that $As(OH)_3$ is still present inside the cells, without undergoing a chemical transformation and is passively effluxed from the cells. These data are corroborated by the observations that i) at pH ~7.3, the rate of complexation of GSH to arsenic is very slow; ii) the rate of passive uptake and efflux of arsenic species in sensitive cells does not depend on GSH level (if arsenic-GSH complex was formed one could have expected different rates of arsenic accumulation inside the GSH-depleted or GSH-rich sensitive cells). So we concluded that within the first 1-2 hours the amount of arsenic-glutathione complex, if any, is very low. Now as time elapses, one observes a slow increase of the arsenic intracellular concentration which becomes higher than the extracellular arsenic concentration. One very likely interpretation is that the arsenic accumulation is due to the transformation of the permeant $As(OH)_3$ species into another one which is unable to diffuse freely through the membrane such as an As-glutathione complex or arsenic binding to thiol-containing proteins. All this reasoning helds for antimony.

The important point is that a negligible amount, if any, of As-GSH complex is formed during the first 1-2 hours. The arsenic concentration is lower in resistant cells than in sensitive cells, thus, the arsenic species is pumped out by MRP1. This shows that $As(OH)_3$ species is the substrate for MRP1.

In addition to the observation that there is no active efflux in GSH-depleted cells, we suggest that $As(OH)_3$ could be co-transported with GSH. In addition, 2 μM MK571 are able to inhibit the 50% of MRP1-mediated efflux of $As(OH)_3$ and this is exactly the concentration required to inhibit 50% of MRP1-mediated efflux of GSH /12/.

### CONCLUSIONS

Over the past 15 years, considerable advances have been made in knowledge of multidrug efflux systems. Exponentially growing data indicate that these systems are widespread both in prokaryotes and eukaryotes and suggest that they have a dual physiological function, first as secretion machineries for cellular products and, second, as defense mechanisms against harmful substances present in the environment. It is intriguing to speculate on the origins of resistance mechanisms. For instance, considering that toxic metal ions were present in the primordial oceans, resistance to metals probably arose long before antibiotic resistances. Then, presented with new environmental challenges, there would have been selective pressure on eukaryotes to evolve additional mechanisms. Thus, cellular overexpression of MRP1 confers a resistance phenotype mainly restricted to antimonial and arsenical salts and not to other cytotoxic metals such as chromium, aluminium, cobalt, platinum. Such a mechanism of resistance may be important for clinical efficiency of arsenical salts used in the treatment of some leukemias

### BIBLIOGRAPHY

1. M. Gottesman, I. Pastan and S. Ambudkar, *Curr. Biol.* **6**, 610 (1996).
2. F. Sharom, *J. Membrane Biol.* **160**, 161 (1997).
3. P. Borst and M. Ouellette, *Annu. Rev. Microbiol.* **49**, 427 (1995).
4. H. Bolhuis, H. vanVeen, B. Poolman, A. Driessen and W. Konings, *FEMS Microbiol. Rev.* **21**, 55 (1997).
5. P. Borst, R. Evers, M. Kool and J. Wijnholds, *J. Nat. Cancer Inst.* **92**, 1295 (2000).
6. C. Higgins , *Annu. Rev. Cell Biol.* **8**, 67 (1992).
7. D. Loe, K. Almquist, R. Deeley and S. Cole, *J. Biol. Chem.* **271**, 675 (1996).
8. I. Leier, G. Jedlitschky, U. Buchholz, S. Cole, R. Deeley and D. Keppler *J. Biol. Chem.* **269**, 27807 (1994).
9. C. Versantvoort, H. Broxterman, T. Bagrij, R. Scheper and P. Twentyman, *Br. J. Cancer* **72**, 82 (1995).
10. J. Renes, E. De Vries, E. Nuenhuis, P. Jansen. and M. Muller, *Br. J. Pharmacol.* **126**, 681 (1999).

11. C. Marbeuf-Gueye, H. Broxterman, F. Dubru, W. Priebe and A. Garnier-Suillerot, *Mol. Pharmacol.* **53**, 141 (1998).
12. M. Salerno and A. Garnier-Suillerot, *Eur. J. Pharmacol.* **421**, 1 (2001).
13. P. Enterline, R. Day and G. Marsh, *Occup. Environ. Med.* **52**, 28 (1995).
14. H. Chiou, W. Huang, C. Su, S. Chang, Y. Hsu and C. Chen, *Stroke* **28**, 1717 (1997).
15. C. Hopenhayn-Rich, M. Biggs and A. Smith, *Int. J. Epidemiol.* **27**, 561 (1998).
16. D. Lewis, J. Southwick, R. Ouellet-Hellstrom, J. Rench and R. Calderon, *Environ. Health Perspect.* **107**, 59 (1999).
17. E. Crecelius, *Environ. Health Perspect.* **19**, 147 (1977).
18. T. Smith, E. Crecelius and J. Reading JC. *Environ. Health Perspect.* **19**, 89 (1977).
19. H. Yamauch and Y. Yamamura, *Ind. Hyg.* **17**, 79 (1979)..
20. W. Cullen, B. McBride and J. Reglinskli, *J. Inorg. Biochem.* **21**, 179 (1984).
21. W. Cullen, B. McBride, H. Manji, A. Pickett and J. Reglinsky, *Appl. Organomet. Chem.* **3**, 71 (1989).
22. M. Mass, A.Tennant, B. Roop, W. Cullen, M. Styblo, D. Thomas and A. Kigerman, *Chem. Res. Toxicol.* **14**, 355 (2001).
23. M. Wei, H. Wanibuchi, S. Yamamoto, W. Li and H. Fukushima, *Carcinogenesis* **20**, 1873 (1999).
24. N. Scott, K. Hatelid, N. MacKenzie and D. Cater, *Chem. Res. Toxicol.* **6**, 102 (1993)..
25. M. Delnomdedieu, M. Basti, J. Otvos and J. Thomas, *Chem.- Biol. Inter.* **90**, 139 (1994).
26. R. Zakharyan and H. Aposhian, *Chem. Res. Toxicol.* **12**, 1278 (1999).
27. T. Radabaugh and H. Aposhian,. *Chem. Res. Toxicol.* **13**, 26 (2000).
28. D. Rabenstein, Metal complexes of glutathione and their biological significance. In: *Glutathione: Chemical, Biochemical and Medicinal Aspects* (D. Dolphin, O. Avramomic and R. Poulson eds) pp 147-186. John Wiley & Sons, New York (1998).
29. M. Salerno, M. Petroutsa and A. Garnier-Suillerot, *J. Bioenerg. Biomembr.* **34**, 135 (2002).
30. S. Kala, M. Neely, G. Kala , C. Prater, D. Atwood, J. Rice and M. Liberman, *J. Biol. Chem.* **275**, 33404 (2000).
31. Z. Gregus, A. Gyurasics and I. Csanaky, *Toxicol. Sci* **56**, 18 (2000).
32. H. Aposhian, *Pharmacol. Toxicol.* **37**, 397 (1997).
33. R. Mukhopadhyay, S. Dey, N. Xu, D. Gage, J. Lightbody, M. Ouellette and B. Rosen, *Proc. Natl. Acad. Sci. USA* **93**, 10383 (1996).
34. S. Dey and B. Rosen in: *Drug Transport in Antimicrobial and Anticancer Chemotherapy* (N. Georgopapadakoi, ed.) pp.103-132, Dekker (1995).
35. B. Rosen, *Trends Microbiol.* **208**, 207 (1999).
36. R. Ashford, P. Desjeux and P. de Raadt, *Parasitol. Today* **8**, 104 (1992).
37. S.Reiner, R. and R. Locksley, Annu. Rev. *Immunol.* **13**, 151 (1995).
38. J. Berman, D. Waddell and B. Hanson, *Antimicrob. Agents Chemother.* **39**, 1243 (1985).
39. W. Roberts, J. Berman and P. Rainey, *Antimicrob. Agents Chemother.* **39**, 1234 (1995).
40. B. Papadopoulou, C. Kundig, A. Singh and M. Ouellette, *Drug Resistance Updates* **1**, 266 (1998).
41. J. Jackson, J. Tally and W. Ellis, *Am. J. Trop. Med. Hyg.* 43, 464 (1990).

42. M. Ouellette and B. Papadopoulou, *Parasitol. Today* **9**, 150 (1993).
43. P. Shaked-Mishan, N. Ulrich, M. Ephros and D. Zilberstein, *J. Biol. Chem.* **276**, 3971 (2001).
44. D. Legar, S. Cayer, A. Singh, D. Richard, B. Papadopoulou and M. Ouellette, *J. Bioenerg. Biomembrane* **33**, 469 (2001).
45. M. Wintrobe, *Clinical Hematology*, 3rd edn. Henry Kimpton, London, pp 723, 866,.917 (1951).
46. Z. Shen, G. Chen, J. Ni, X. Li, S. Xion, Q. Qiu, J. Zhu, W. Tang, G. Sun, K. Yang, Y. Chen, Z. Fang, Y. Wang, J. Ma, P. Zhang, T. Zhang, S. Chen and Z. Wang, *Blood* 9, 3354 (1997).
47. A. Bode and Z. Dong, *Drug Resistance Update* **3**, 21 (2000).
48. S. Cole, K. Sparks, K. Fraser, D. Loe, C. Grant, G. Wilson and R. Deeley, *Cancer Res.* **54**, 5902 (1994).
49. B. Stride, C. Grant, D. Loe, D. Hipfner, S. Cole and R. Deeley, *Mol. Pharmacol.* **52**, 344(1997).
50. L. Vernhet, N. Allain, C. Bardia, J.-P. Anger and O. Fardel, *Toxicol.***142**, 127 (2000).
51. L. Vernhet, A. Courtois, L. Payen, N. Allain, J.P. Anger, A. Guillouzou and O. Fardel, *FEBS Lett.* **443**, 321 (1999).
52. Z. Chen, Z. Wang. and S. Chen, *Pharmacol. Ther.* **76**, 141 (1997)..
53. D. Loe, R. Deeley and S. Cole,(1998). *Cancer Res.* **58**, 5130 (1998).
54. Z. Gregus and A. Gyurasics, *Biochem. Pharmacol.* **59**, 1375 (2000).
55. A. Gyurasics, L. Koszorus, F. Varga and Z. Gregus, Z., *Biochem.* Pharmacol. **44**, 1275 (1992).
56. G. Zaman, J. Lankelma, O., Vantellingen, J. Beijnen, H. Dekker, C. Paulusma, R. Oudeelferink, F. Baas and P. Borst, *Proc. Natl. Acad. Sci.*, USA **92**, 7690 (1995)

